# Surface Modification of Fe-Based Perovskite Oxide via Sr_0.95_Ce_0.05_CoO_3−δ_ Infiltration: A Strategy for Thermochemical Stability

**DOI:** 10.3390/nano15120934

**Published:** 2025-06-16

**Authors:** Taeheun Lim, Heesoo Lee

**Affiliations:** School of Materials Science and Engineering, Pusan National University, Busan 46241, Republic of Korea; taeheunlim@pusan.ac.kr

**Keywords:** iron-based perovskite, cobalt-based perovskite, surface modification, infiltration, composite perovskite oxide, thermochemical stability

## Abstract

Cobalt-based perovskite oxides exhibit remarkable catalytic activity owing to abundant oxygen vacancies and mixed ionic–electronic conductivity, but they suffer from structural instability. In contrast, iron-based perovskite oxides are thermochemically stable under oxidizing and reducing conditions but are catalytically limited. To combine these complementary properties, a composite perovskite oxide was designed and prepared by infiltrating Sr_0.95_Ce_0.05_CoO_3−δ_ (SCC) into Ba_0.5_Sr_0.5_Fe_0.8_Cu_0.2_O_3−δ_ (BSFC). The SCC precursor solution was dropwise applied to a BSFC|SDC|BSFC symmetric cell and heat treated. Surface morphology and compositional analyses confirmed the distribution of SCC nanoparticles on the BSFC surface. High-temperature X-ray diffraction and Rietveld refinement results revealed that both BSFC and SCC retained the cubic perovskite structure (space group Pm-3m) at room temperature. No phase transition or secondary phase formation was observed during heating from 200 to 800 °C, and the peak shifts are attributed to thermal expansion and possible oxygen loss at elevated temperatures. Upon cooling, the diffraction patterns returned to their initial state, confirming a high-temperature structural stability. XPS analysis showed an increase in the satellite peak intensity associated with Fe^3+^ after SCC infiltration, and the average oxidation state of Fe decreased from 3.52 (BSFC) to 3.49 (composite perovskite oxide). The O 1s spectra revealed a higher relative content of surface-adsorbed oxygen species in the composite, indicating increased oxygen vacancy formation.

## 1. Introduction

Perovskite oxides are widely studied for their tunable functionalities, such as electronic/ionic conduction, ferromagnetism, and ferroelectricity, based on their flexible crystal structures and compositions [1,2]. These properties can be finely controlled via A- and B-site ion substitution, enabling applications in catalysts, sensors, and electronic ceramics [3,4,5].

Cobalt-based perovskite oxides, including La_0.6_Sr_0.4_Co_0.2_Fe_0.8_O_3−δ_ (LSCF) and Ba_0.5_Sr_0.5_Co_0.8_Fe_0.2_O_3−δ_ (BSCF), show excellent oxygen reduction reaction (ORR) activity due to high oxygen vacancy concentrations and mixed ionic–electronic conductivity [6,7]. However, the high-temperature phase stability is degraded under redox conditions due to covalence/spin transitions and oxygen vacancy formation [8,9]. For example, cubic BSCF gradually transforms into hexagonal and lamellar phases at 500–800 °C in air [10,11]. In contrast, iron-based perovskite oxides such as BaFeO_3_ and SrFeO_3_ offer better thermal stability due to the stable electronic structure of Fe ions [12], but lower electrochemical performance limits their single-phase cathode use [13].

Surface modification via infiltration offers a promising route to enhance performance in energy devices like SOFCs and SOECs [14]. Infiltration introduces functional catalyst layers onto cathode surfaces, leading to changes in surface chemistry and thereby enhancing surface activity [15]. While LSM cathodes with GDC or SDC infiltration and LSCF with cobalt-based infiltration have been reported, iron-based cathodes modified with cobalt-based oxides have not been widely studied [15,16,17,18].

Among the cobalt-based oxides, Sr_0.95_Ce_0.05_CoO_3−δ_ (SCC) was selected as the infiltrate due to its improved high-temperature phase stability and electrochemical activity resulting from Ce^4+^ doping. Ce doping into SrCoO_3−δ_ has been reported to stabilize the cubic perovskite structure across a wide temperature range, suppressing the formation of hexagonal or orthorhombic phases, which typically degrade electrochemical performance [19]. Several studies have shown that Ce^4+^ substitution enhances electrical conductivity, reduces polarization resistance, and promotes long-term stability [20,21,22]. Based on these advantages, SCC was employed to modify the surface of BSFC.

We confirmed the potential of Ba_0.5_Sr_0.5_Fe_0.8_Cu_0.2_O_3−δ_ (BSFC) as a cathode material through Cu doping [23]. In this study, the high-temperature phase stability and surface chemical characteristics of the composite perovskite oxide were investigated by introducing SCC to the surface of BSFC via an infiltration process.

## 2. Materials and Methods

### 2.1. Synthesis of Ba_0.5_Sr_0.5_Fe_0.8_Cu_0.2_O_3−δ_ (BSFC) and Sr_0.95_Ce_0.05_CoO_3−δ_ (SCC) Perovskite Powder

BSFC and SCC powders were synthesized using the ethylenediaminetetraacetic acid (EDTA) citrate complexing process, ECCP. Stoichiometric amounts of metal nitrates—Ba(NO_3_)_2_ (≥99%, Sigma-Aldrich, St. Louis, MO, USA), Sr(NO_3_)_2_ (≥98%, Alfa Aesar, Ward Hill, MA, USA), Fe(NO_3_)_3_·9H_2_O (≥98%, Sigma-Aldrich, St. Louis, MO, USA), and Cu(NO_3_)_2_·2.5H_2_O (≥98%, Alfa Aesar, Ward Hill, MA, USA) for BSFC; Sr(NO_3_)_2_ (≥98%, Alfa Aesar, Ward Hill, MA, USA), Ce(NO_3_)_3_·6H_2_O (≥99.9%, Daejung, Siheung-si, Republic of Korea), and Co(NO_3_)_3_·6H_2_O (≥98%, Sigma-Aldrich, St. Louis, MO, USA) for SCC—were dissolved in distilled water. EDTA (99.5%, Alfa Aesar, Ward Hill, MA, USA) was dissolved in 1 N NH_4_OH to form NH_3_–EDTA buffer solution, which was added to the metal precursor solution with citric acid monohydrate (metal ions/EDTA/citric acid = 2:1:1). The mixture was stirred at 60 °C for 12 h to form a sol. For BSFC, the sol was preheated at 300 °C for 2 h and calcined in air at 700 °C and 900 °C for 5 h each. For SCC, the sol was sequentially heated at 150 °C and 250 °C for 2 h each, then calcined at 1100 °C for 12 h.

### 2.2. Surface Modification of BSFC with SCC Infiltration

**Figure 1:**
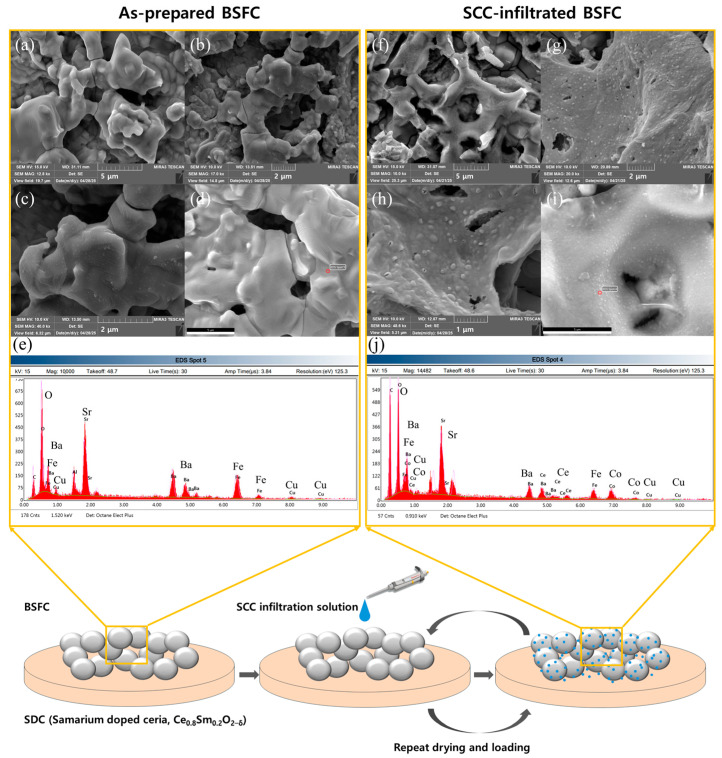
Schematic illustration of infiltration process with FE-SEM and EDS results for as-prepared BSFC (**a**–**e**) and SCC-infiltrated BSFC (**f**–**j**).

A 0.05M SCC precursor solution was prepared in 100 mL of mixed solvent (56 wt.% distilled water and 44 wt.% ethanol), containing 0.5026 g Sr(NO_3_)_2_, 0.05427 g Ce(NO_3_)_3_·6H_2_O, and 0.7275 g Co(NO_3_)_2_·6H_2_O. EDTA (1.4612 g) was added, and pH was adjusted to 7–9 with 1 N NH_4_OH. This solution was dropwise applied (10 μL × 5) onto the BSFC|SDC|BSFC symmetric cell (Figure 1). Each infiltration step was followed by drying at 60 °C for 10 min. The infiltrated specimen was heat-treated at 600 °C for 2 h in air to crystallize the SCC nanoparticles and form a catalytic layer on the BSFC surface.

### 2.3. Characterization

Surface morphology and composition of the infiltrated specimens were observed using field-emission scanning electron microscopy (FE-SEM, MIRA3, TESCAN, Brno, Czech Republic) and energy-dispersive spectrometry (EDS). High-temperature X-ray diffraction (HT-XRD) patterns were collected from 25 °C to 800 °C (20–80°, step 0.039°, scan 1°/min) using a multipurpose X-ray diffractometer (MP-XRD, X’Pert Pro, Malvern Panalytical, Almelo, The Netherlands) and refined using the FullProf software package (ver. 5. 10). High-resolution transmission electron microscopy (HR-TEM, JEOL JEM-2100F, JEOL, Tokyo, Japan) images were analyzed for interplanar spacing using ImageJ (ver. 1.53t). X-ray photoelectron spectroscopy (XPS, Thermo Fisher Scientific, Waltham, MA, USA, Al Kα) was used for surface chemical analysis. XPS spectra were corrected with C 1s (B.E = 284.6 eV) and were fitted using a Gaussian peak shape after standard Tougaard background correction.

For surface morphology and chemical composition, FE-SEM and EDS analyses were performed directly on the surface of the BSFC|SDC|BSFC symmetric cell after SCC infiltration and heat treatment. HT-XRD, HR-TEM, and XPS analyses were conducted using a powder (BSFC+SCC) obtained by gently scraping the infiltrated layer from the symmetric cell. Rietveld refinement was performed using the room-temperature XRD patterns of BSFC and SCC powders synthesized via the ECCP method, as described in Section 2.1.

## 3. Results and Discussion

Sr_0.95_Ce_0.05_CoO_3−δ_ (SCC) infiltration was conducted to investigate its effects on the surface morphology and composition of Ba_0.5_Sr_0.5_Fe_0.8_Cu_0.2_O_3−δ_ (BSFC). Figure 1 presents a schematic diagram of the infiltration process for synthesizing the composite perovskite oxide (BSFC+SCC), along with the microstructural and compositional differences before and after surface modification. The as-prepared BSFC exhibited smooth grain boundaries and a porous structure, and EDS analysis confirmed the presence of Ba, Sr, Fe, Cu, and O elements. In contrast, the SCC-infiltrated BSFC showed distributed nanoscale particles on the surface, and the presence of Co and Ce was confirmed by EDS analysis (see Figure S1 in the Supplementary Materials).

**Figure 2:**
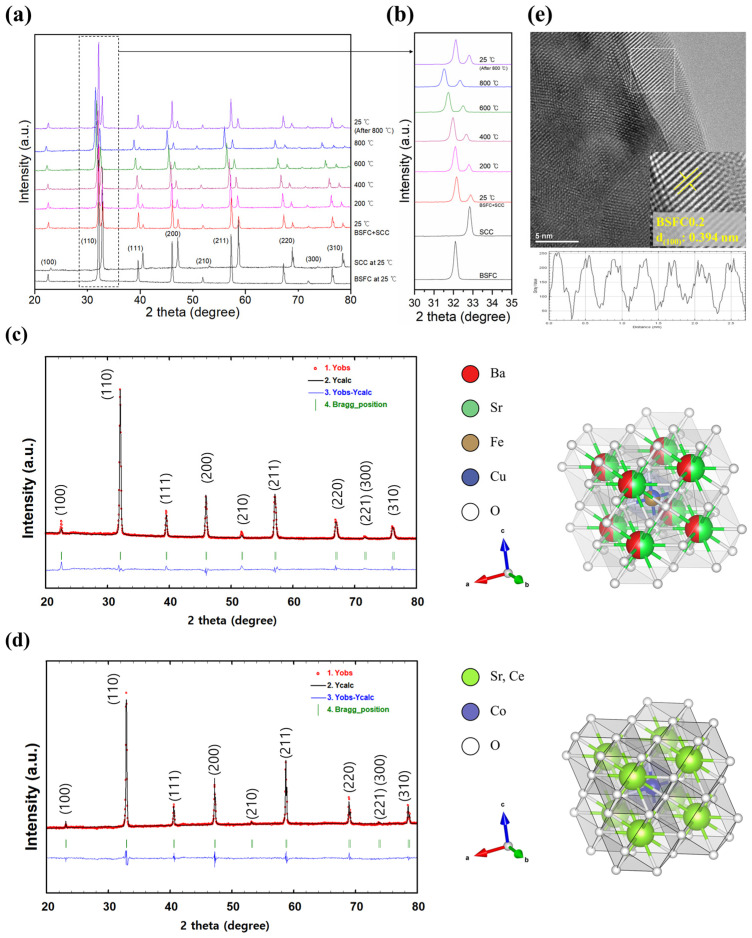
(**a**) In situ HT-XRD patterns of BSFC, SCC at room temperature, and BSFC+SCC at different temperatures. (**b**) The magnification of the main peaks. Rietveld refinement of (**c**) BSFC and (**d**) SCC from XRD data at room temperature (λ = 1.5406 Å) with the insets for refined structure of BSFC and SCC perovskite. (**e**) HR-TEM image of BSFC+SCC composite.

High-temperature X-ray diffraction (HT-XRD) analysis was performed to evaluate the high-temperature phase stability and crystal structure. Figure 2a displays the room-temperature XRD patterns of BSFC and SCC synthesized via the ECCP method, as well as the in situ HT-XRD patterns of the composite sample (BSFC+SCC) over the temperature range of 25–800 °C. Figure 2c,d shows Rietveld refinement of the room-temperature XRD data. The lattice parameters of BSFC and SCC were 3.9492 Å and 3.8490 Å, respectively, and the observed diffraction peaks corresponded to the (100), (110), (111), (200), and (210) planes, characteristic of a cubic perovskite structure with space group Pm-3m (Table 1).

The χ^2^ values obtained from the Rietveld refinement were relatively high, with 6.87 for BSFC and 4.79 for SCC. This is attributed to slight lattice distortions not fully captured by the simple cubic (Pm-3m) structural model [24,25]. In particular, weak peak splitting observed in the high-angle region may indicate local deviations from ideal cubic symmetry. These structural features likely contributed to the increased χ^2^ values.

BSFC+SCC showed no phase transition or secondary phase formation during heating, apart from peak shifts attributed to thermal expansion and possible oxygen loss. The oxygen non-stoichiometry (δ) at high temperatures may also contribute to the lattice expansion through oxygen vacancy formation and B-site cation reduction. This interpretation is consistent with previous studies reporting oxygen loss and cation reduction in BSFC and SCC at temperatures above 600 °C and 400 °C, respectively [19,26]. After cooling, the diffraction patterns returned to their initial state, indicating that both phases coexist in a structurally stable manner from high temperatures down to room temperature. As shown in the enlarged (110) peak region in the 30–35° range (Figure 2b), the peaks from BSFC and SCC remained well separated, with no evidence of peak merging or formation of new peaks after heating to 800 °C, supporting the absence of structural interference caused by the infiltration. Figure 2e shows an HR-TEM image of the BSFC+SCC composite, in which a lattice fringe with a spacing of approximately 0.394 nm corresponding to the (100) plane of BSFC was observed.

**Figure 3:**
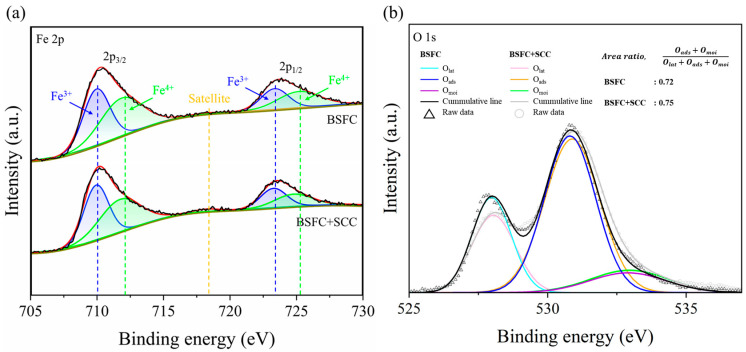
The XPS spectra of (**a**) Fe 2p orbital and (**b**) O 1s orbital for BSFC and BSFC+SCC.

XPS analysis was conducted to investigate the changes in the electronic structure of Fe induced by surface modification (see Figure S2). Figure 3a presents the Fe 2p XPS spectra of BSFC and BSFC+SCC, along with their fitting results. Both spectra were deconvoluted into components corresponding to Fe^3+^, Fe^4+^, and satellite peak. The Fe^3+^ peaks were observed at approximately 709.96 eV and 723.36 eV for BSFC, and 709.93 eV and 723.27 eV for BSFC+SCC. The Fe^4+^ peaks appeared at 711.83 eV and 725.18 eV for BSFC and at 711.72 eV and 724.78 eV for BSFC+SCC. A satellite peak near 718.24 eV supports the presence of a mixed valence state of Fe [27,28,29]. Notably, the intensity of the satellite peak increased in the BSFC+SCC sample compared to BSFC, suggesting a higher relative concentration of Fe^3+^ due to SCC infiltration. According to the fitting results in Table 2, the average oxidation state of Fe decreased from 3.52 in BSFC to 3.49 in BSFC+SCC, indicating that partial reduction from Fe^4+^ to Fe^3+^ occurred through chemical interaction with SCC.

As shown in Figure 3b, the O 1s peak was deconvolved into three components located at 528.0 eV (lattice oxygen, O_lat_), 530.8 eV (adsorbed oxygen, O_ads_), and 532.9 eV (surface moisture, O_moi_). In the ABO_3_ perovskites, the content of O_ads_ and O_moi_ corresponds to the oxygen vacancy concentration [30,31]. The relative content of O_ads_ and O_moi_, defined as O_ads_ + O_moi_/O_lat_ + O_ads_ + O_moi_, is calculated by integrating the peak area of the XPS spectra (Table 3). The relative content of adsorbed oxygen and surface moisture increased from 0.72 in BSFC to 0.75 in BSFC+SCC. This increase in surface-adsorbed oxygen species suggests the presence of more oxygen vacancies in the BSFC+SCC sample, which may facilitate the oxygen ionic conduction and accelerate the rate of oxygen reduction reaction [32,33].

These results suggest that SCC infiltration alters the Fe valence state while maintaining the structural stability of BSFC. However, it does not address electrochemical performance and redox cycling stability, which is a critical factor under actual SOFC operating conditions. Therefore, further studies will be necessary to evaluate the electrochemical performance and structural robustness of the composite under repeated oxidation–reduction environments.

## 4. Conclusions

We designed and prepared a composite perovskite oxide (BSFC+SCC) by infiltrating Sr_0.95_Ce_0.05_CoO_3−δ_ (SCC) into an iron-based perovskite oxide, Ba_0.5_Sr_0.5_Fe_0.8_Cu_0.2_O_3−δ_ (BSFC), and investigated the effects of surface modification on the structural and chemical properties. Surface morphology and compositional analyses revealed that the infiltrated SCC existed as distributed nanoparticles on the BSFC surface. According to HT-XRD analysis, both BSFC and SCC exhibited a cubic perovskite structure (space group Pm-3m) with lattice parameters of 3.9492 Å and 3.8490 Å, respectively. The BSFC+SCC composite remained structurally stable without phase transition or secondary phase formation throughout the heating and cooling process from 25 to 800 °C. TEM analysis also confirmed that the lattice structure of BSFC was retained after infiltration, with an interplanar spacing corresponding to the (100) plane observed. XPS analysis showed an increased intensity of the Fe 2p satellite peak following SCC infiltration, and the average oxidation state of Fe decreased from 3.52 (BSFC) to 3.49 (BSFC+SCC). The O 1s spectra revealed a higher relative content of surface-adsorbed oxygen species in the BSFC+SCC composite, suggesting enhanced oxygen vacancy formation. This oxygen deficiency may enhance oxygen ion transport and catalytic activity for the oxygen reduction reaction (ORR). These results indicate that SCC infiltration induced changes in the surface chemical composition while maintaining the structural stability of BSFC.

## Figures and Tables

**Table 1:** Structural parameters of the BSFC and SCC calculated by Rietveld refinement of the room-temperature XRD data.

Parameters	Composition	
	BSFC	SCC
a = b = c [Å]	3.9492(5)	3.8490(2)
Volume [Å^3^]	61.593(5)	57.024(3)
Space group	Pm-3m	Pm-3m
R_p_ [%]	3.05	3.32
R_wp_ [%]	5.87	4.53
R_exp_ [%]	2.24	2.07
χ^2^	6.87	4.79

**Table 2:** Fitting results of the Fe 2p orbital XPS spectra of BSFC and BSFC+SCC.

Sample	Fe^3+^ (%)	Fe^4+^ (%)	Average Oxidation State
BSFC	47.77	52.23	3.52 (±0.0015)
BSFC+SCC	50.82	49.18	3.49 (±0.0017)

**Table 3:** Fitting results of the O1s orbital XPS spectra of BSFC and BSFC+SCC.

Sample	O_lat_		O_ads_		O_moi_		Area Ratio of
	Peak (eV)	Area	Peak	Area	Peak	Area	O_ads_ + O_moi_/O_lat_ + O_ads_ + O_moi_
BSFC	527.98	25,270.25	530.81	54,085.33	532.90	10,093.92	0.72
BSFC+SCC	528.04	22,827.22	530.88	57,041.56	532.96	11,667.61	0.75

## Data Availability

Data are contained within the article and Supplementary Materials.

## Supplementary Materials

The following supporting information can be downloaded at https://www.mdpi.com/article/10.3390/nano15120934/s1: Figure S1: (a–d, f–i) Field emission scanning electron microscopy images with (e, j) energy dispersive spectrometry results of Ba_0.5_Sr_0.5_Fe_0.8_Cu_0.2_O_3−δ_ and Sr_0.95_Ce_0.05_CoO_3−δ_ nanocomposite. Figure S2: The XPS spectra of Ba_0.5_Sr_0.5_Fe_0.8_Cu_0.2_O_3−δ_ (BSFC) and Sr_0.95_Ce_0.05_CoO_3−δ_-infiltrated Ba_0.5_Sr_0.5_Fe_0.8_Cu_0.2_O_3−δ_ (BSFC+SCC) in the range of (a) 0 eV–1200 eV.
