# Supplementary material for: Surface Modification of Fe-Based Perovskite Oxide via Sr0.95Ce0.05CoO3−δ Infiltration: A Strategy for Thermochemical Stability
Source: Nanomaterials (Basel). 2025 Jun 16;15(12):934. doi: 10.3390/nano15120934 (PMC12196367; doi:10.3390/nano15120934)
Supplement: Supplementary file 1 [file nanomaterials-15-00934-s001.zip › nanomaterials-3694615-supplementary.pdf]

nanomaterials

Supplementary Material

Surface Modification of Fe-based Perovskite Oxide via $Sr_{0.95}Ce_{0.05}CoO_{3-\delta}$ Infiltration: A Strategy for Thermochemical Stability

Taeheun Lim and Heesoo Lee *

School of Materials Science and Engineering, Pusan National University, Busan 46241, Republic of Korea; taeheunlim@pusan.ac.kr

* Correspondence: Correspondence: heesoo@pusan.ac.kr; Tel: +82-51-510-2388

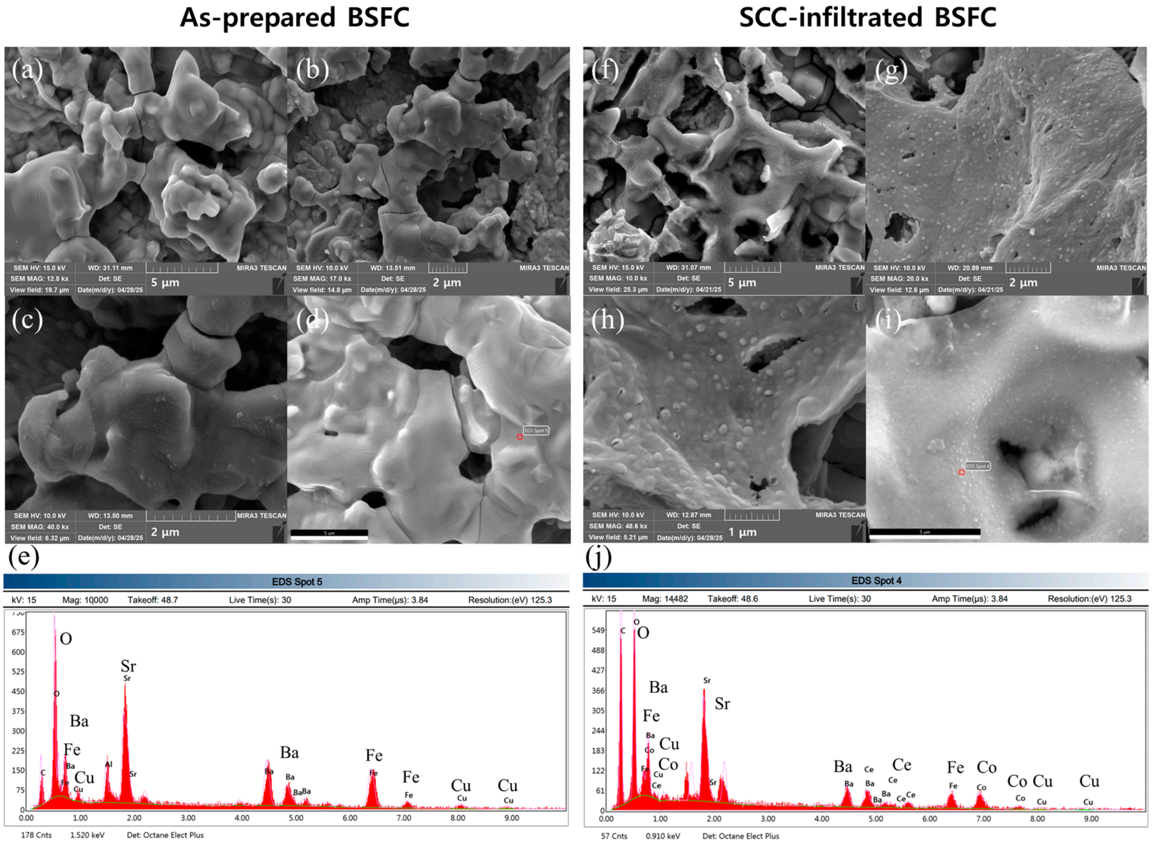

Figure S1. (a–d, f–i) Field emission scanning electron microscopy images with (e, j) energy dispersive spectrometry results of $Ba_{0.5}Sr_{0.5}Fe_{0.8}Cu_{0.2}O_{3-\delta}$ and $Sr_{0.95}Ce_{0.05}CoO_{3-\delta}$ nanocomposite.

$Ba_{0.5}Sr_{0.5}Fe_{0.8}Cu_{0.2}O_{3-\delta}$ (BSFC) before and after $Sr_{0.95}Ce_{0.05}CoO_{3-\delta}$ (SCC) infiltration were examined using SEM–EDS. As shown in Figure S1a–d, the pristine BSFC exhibited porous morphology with smooth grain boundaries and well-developed necking between

 https://doi.org/10.3390/nano15120934

particles, forming a suitable pore structure for capillary-driven infiltration. After SCC infiltration and heat treatment, the surface morphology changed significantly (Figure S1f–i). Uniformly distributed nanoparticles were observed on the BSFC surface in Figure S1h and i, which are attributed to the crystallization of SCC precursor during 600 °C annealing. These particles were absent in the pristine sample, indicating the introduction of SCC onto the BSFC surface.

In addition, EDS point analysis confirmed the surface composition before and after infiltration. The pristine BSFC showed signals corresponding to Ba, Sr, Fe, Cu, and O (Figure S1e), consistent with its composition. In contrast, the infiltrated sample exhibited additional peaks for Co and Ce (Figure S1j), which correspond to incorporation of SCC onto the BSFC surface.

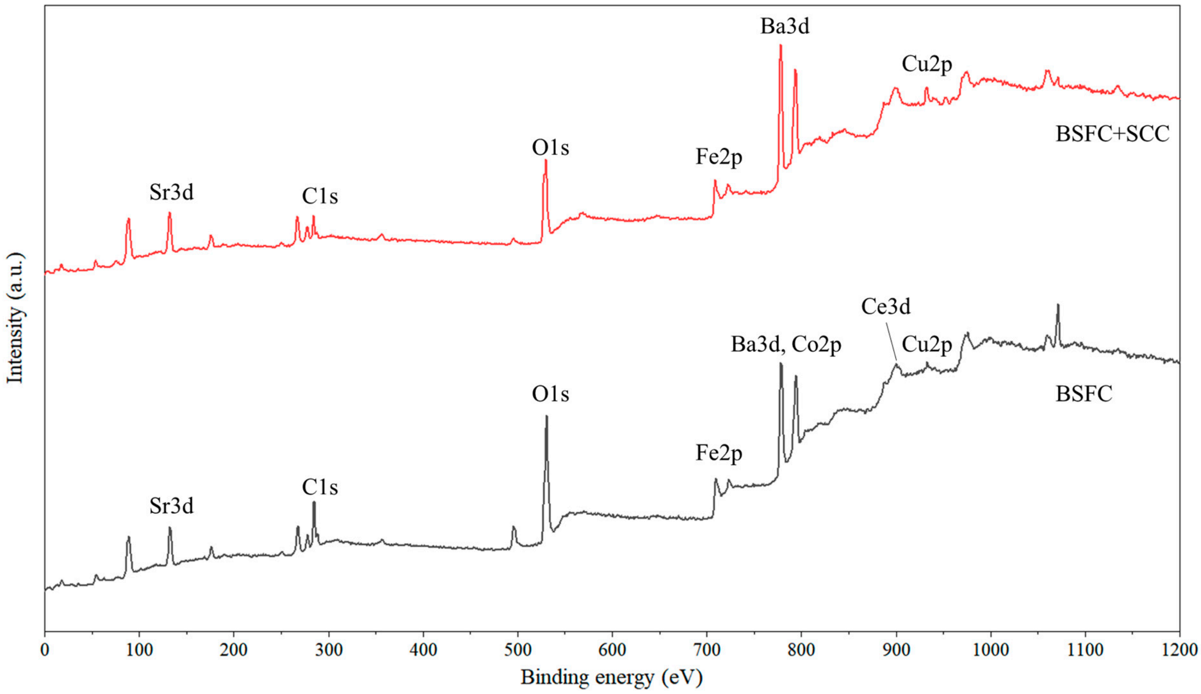

Figure S2. The XPS spectra of $Ba_{0.5}Sr_{0.5}Fe_{0.8}Cu_{0.2}O_{3-\delta}$ (BSFC) and $Sr_{0.95}Ce_{0.05}CoO_{3-\delta}$-infiltrated $Ba_{0.5}Sr_{0.5}Fe_{0.8}Cu_{0.2}O_{3-\delta}$ (BSFC+SCC) in the range of (a) 0 eV–1200 eV.
